# Obstetric outcomes in pregnant COVID-19 women: the imbalance of von Willebrand factor and ADAMTS13 axis

**DOI:** 10.1186/s12884-022-04405-8

**Published:** 2022-02-21

**Authors:** Elvira Grandone, Antonella Vimercati, Felice Sorrentino, Donatella Colaizzo, Angelo Ostuni, Oronzo Ceci, Manuela Capozza, Giovanni Tiscia, Antonio De Laurenzo, Mario Mastroianno, Filomena Cappucci, Lucia Fischetti, Maurizio Margaglione, Ettore Cicinelli, Luigi Nappi

**Affiliations:** 1 Thrombosis and Haemostasis Unit, Fondazione I.R.C.C.S. “Casa Sollievo della Sofferenza”, Viale Cappuccini, 71013 Foggia, S. Giovanni Rotondo Italy; 2 Ob/Gyn Department of The First I.M. Sechenov Moscow State Medical University, Moscow, Russia; 3 Ob/Gyn Institute, Department of Medical and Surgical Sciences, University of Foggia, Foggia, Italy; 4 Ob/Gyn Institute, Department of Biomedical Sciences and Human Oncology, University of Bari “Aldo Moro”, Bari, Italy; 5 Immunohematology and Transfusion Medicine Service, Azienda Ospedaliero-Universitaria Consorziale Policlinico di Bari, University of Bari “Aldo Moro”, Bari, Italy; 6 Struttura Regionale Coordinamento Puglia, Bari, Italy; 7 Neonatal Intensive Care Unit, Department of Biomedical Sciences and Human Oncology, University of Bari “Aldo Moro”, Bari, Italy; 8 Scientific Direction, Fondazione I.R.C.C.S. “Casa Sollievo della Sofferenza”, Foggia, S. Giovanni Rotondo Italy; 9 Medical Genetics, University of Foggia, Foggia, Italy

**Keywords:** ADAMTS13, COVID-19, von Willebrand factor, AB0 blood group system, Pregnancy

## Abstract

**Background:**

Thrombotic microangiopathy has been invoked as one of the most important mechanisms of damage in COVID-19 patients. Protease ADAMTS13 is a marker of microangiopathy responsible for controlling von Willebrand multimers size. Von Willebrand factor/ADAMTS13 ratio has been found impaired in COVID-19 patients outside pregnancy.

**Methods:**

We prospectively investigated 90 pregnant women admitted to two tertiary academic hospitals in Italy with a laboratory-confirmed diagnosis of SARS-CoV-2 infection. Demographic, clinical information and routine laboratory data were collected at the hospital admission and until discharge. We investigated whether vonWillebrand /ADAMTS13 axis imbalance is a predictor of adverse outcomes. Logistic regression analysis, which controlled for potential confounders, was performed to evaluate the association between laboratory parameters and clinical outcomes.

**Results:**

Most women (55.6%) were parae, with median gestational age at admission of 39 weeks. At hospital admission, 63.3% were asymptomatic for COVID-19 and 24.4% showed more than one sign or symptom of infection. Nulliparae with group O showed Willebrand / ADA MTS-13 ratios significantly lower than non-O, whereas in multiparae this difference was not observed. Logistic regression showed that ratio von Willebrand to ADAMTS13 was significantly and independently associated with preterm delivery (OR 1.9, 95%CI 1.1–3.5).

**Conclusion:**

This study shows an imbalance of vonWillebrand /ADAMTS13 axis in pregnant women with COVID-19, leading to a significantly higher and independent risk of preterm delivery. Monitoring these biomarkers might support decision making process to manage and follow-up pregnancies in this setting.

## Introduction

Coronavirus Disease 2019 (COVID-19) spread rapidly worldwide. The spectrum of clinical manifestations of pregnant women with COVID-19 varies widely from asymptomatic to severe. Indeed, its clinical manifestations range from mild -as common cold- to severe disease- as severe acute respiratory syndrome. Although knowledge on a series of specific aspects has increased rapidly, evidence specifically focused on pregnancy is still limited. Some authors have suggested a higher incidence of foetal -maternal adverse outcomes [1, 2]. Pregnancy complications so far described include miscarriage, stillbirth, foetal distress, premature rupture of membranes, preterm delivery and post-partum haemorrhage [3, 4]. Recent findings show that during pregnancy an impaired expression of IL-1 and TNF-a- variable by pregnancy trimesters- is associated with an increased risk of miscarriage and preterm delivery [4]. The pathophysiological events behind the increased risk of obstetric complications are basically the cytokine storm and the activation of circulating cells as macrophages and T lymphocytes and endothelial cells [5, 6].

SARS-CoV-2, responsible for COVID-19, likely promotes endotheliitis in different organs and tissues, leading to endothelial damage [7] and, in turn, thrombotic microangiopathy associated to the most threatening consequences of the infection [8–10].

Thrombotic microangiopathy is characterised by microangiopathic haemolytic anaemia, thrombocytopenia, and organ damage. It can manifest itself in a large number of pathological conditions [thrombotic thrombocytopenic purpura (TTP), atypical haemolytic uremic syndrome, infections, tumors] or be associated with a history of transplantation and the use of drugs [11]. Thrombotic microangiopathies require prompt treatment, as it reduces mortality. For example, TTP, if immediately treated with the plasma exchange (PEX) technique, leads to a reduction in mortality risk from about 90 to 20% [12, 13]. An important role, in the context of thrombotic microangiopathies, is played by the ADAMTS13 (*a d*isintegrin *a*nd *m*etalloproteinase with a *t*hrombo*s*pondin type 1 motif, member *13*) plasma protease. Under physiological conditions, ADAMTS13 cleaves von Willebrand factor (vWF) into monomers, eliminating from the circulation the multimers of vWF which, otherwise, cause platelet thrombi especially in small vessels. The dosage of ADAMTS-3 therefore facilitates the differential diagnosis of thrombotic microangiopathies. A picture of thrombotic microangiopathy can also occur during pregnancy when HELLP syndrome or preeclampsia occur. Indeed, thrombotic microangiopathy with activation of endothelial cells and multi-organ involvement characterise these severe complications of pregnancy. In these conditions, vWF/ADAMTS13 axis has been explored with sometimes conflicting results [14].

Paucity of data is available on ADAMTS13 fluctuations during physiological pregnancies [15, 16]. ADAMTS13 activity decreases progressively from 12 to 16 weeks up to the end of early puerperium (mean 52%, range 22–89) and increases slightly thereafter [16].

Recently, it has been documented that thrombotic microangiopathy can occur, especially in the most severe forms, in the context of SARS-CoV-2 infection and that reduced levels of ADAMTS13 are associated with a poor prognosis [8, 17]. In pregnant women with COVID-19 it might be clinically useful to correlate the protease’s levels with the foetal and maternal outcome.

We present clinical and laboratory features, outcomes and data on vWF ADAMTS 13 axis obtained in a cohort of pregnant women with COVID-19 consecutively admitted to two Academic Hospitals in Southern Italy.

Aims of the present study were to investigate whether FVIII, Willebrand (VWF) Antigen (Ag) and ristocetin-cofactor (VWF:RCo) predicted adverse outcomes in pregnant women with infection by SARS-Cov-2 infection.

## Patients and methods

### Patients

We investigated 90 pregnant women consecutively admitted to two tertiary academic hospitals in the Apulia region between December 2nd 2020 and January 31st 2021 with a laboratory-confirmed diagnosis (i.e., RT-PCR according to the protocol established by the WHO) of SARS-CoV-2 infection. Demographic, clinical information and routine laboratory data were collected at the hospital admission and until discharge.

Demographic data, comorbidities, medications and clinical variables including respiratory support were obtained from clinicians (AV, FS, OC, MC) at time of admission to hospital and during the in-hospital stay.

We collected data on pregnancy and neonatal outcome, including gestational age at delivery, mode of delivery, indication for caesarean delivery, complications, neonatal birthweight, Apgar scores and neonatal intensive care unit (NICU) admission. The date of data cut off for outcomes was February 28th, 2021.

Approval was obtained from the Ethics Committee at the University of Bari. Each patient signed an informed consent to the study and utilisation of data for research purposes. The STROBE guidelines for reporting observational studies in epidemiology were followed.

## Methods

Laboratory assessments consisted of complete blood cell count, liver and renal function, hs C-reactive protein (hsCRP), D-dimer and coagulation tests.

Plasma for ADAMTS13 activity evaluation was collected from whole human blood anticoagulated with 12.9 mM sodium citrate. Samples were centrifuged by using two-step centrifugation (2500 g, 20 min) at 20 °C. Samples were aliquoted and frozen at − 80 °C until analysed.

In all patients, ADAMTS13, vWF antigen (vWF:Ag)/ functional levels were measured on a blood sample obtained within three hospitalization days. The ADAMTS13 activity levels were measured using a chromogenic enzyme-linked immunosorbent assay (ELISA) method (TECHNOZYM ADAMTS13 Activity ELISA Kit, Technoclone, Austria), as previously described [8].

### Definitions

Miscarriage was defined as nonviable intrauterine pregnancy up to 20 weeks’ gestation.

IUFD (Intrauterine Foetal Death) was defined as an otherwise unexplained foetal demise occurred after 20 weeks, as previously defined [18].

Preeclampsia and HELLP syndrome were defined according to ACOG criteria [19].

PPROM (Preterm Premature Rupture of membranes) was defined as the rupture of membranes during pregnancy before 37 weeks’ gestation.

Preterm delivery was defined as the birth of a baby before 37 weeks.

### Statistical analysis

Continuous variables are presented as a median and interquartile range, whereas discrete variables as numbers and percentage. After testing for data normality distribution, Mann–Whitney U test was used to analyse differences in continuous variables.

Logistic regression analysis, which controlled for age, Body Mass Index (BMI), parity, D-Dimer**,** Neutrophils Lymphocytes Ratio (NLR), comorbidities, FVIII, Willebrand RCo and Ag, blood group, haemoglobin values at admission was performed to evaluate the association between laboratory parameters and clinical outcomes.

## Results

Demographic and laboratory features of women enrolled are shown in Tables 1 and 2, respectively. Most (55.6%) were parae, whereas 44.4% were at their first pregnancy. Median gestational age at admission was 39 weeks and the vast majority of them (80%) was near term (Table 1). Overall, 18.9% had at least one miscarriage or one IUFD in the obstetric history.

**Table 1:** Baseline clinical data in the full cohort (*n* = 90)

Variable	Values
***Maternal features***	
Age, median (range) yrs	32 (17–46)
BMI, median (IQR)	28.3 (4.7)
Blood group 0, n (%)	32 (35.6)
Blood group A, n (%)	39 (43.3)
Blood group B, n (%)	14 (15.6)
Blood group AB, n (%)	5 (5.5)
***Gestational age on admission, week***	
Range, median (range)	39 (9–42)
< 13, n (%)	3 (3.3)
14–27, n (%)	3 (3.3)
28–36, n (%)	12 (13.3)
≥ 37, n (%)	72 (80)
***Pre-existing maternal co-morbidities n (%)***	4 (4.4)
***Obstetric history*** **n (%)**	
Nulliparity	40 (44.4)
Previous at-term deliveries	47 (52.2)
Previous preterm deliveries	3 (3.3)
Previous Pregnancy Loss	16 (17.8)
IUFD	1 (1.1)
***COVID-19 Symptoms and signs*** **n (%)**	
No symptoms	57 (63.3)
Fever	6 (6.7)
Cough	3 (3.3)
Sore throat	1 (1.1)
Myalgia	6 (6.3)
Dyspnea	2 (2.2)
More than one	22 (24.4)

**Table 2:** Baseline laboratory data in the full cohort

***Laboratory data,*** median (IQR)		Reference values
Prothrombin Time (International Normalized Ratio),	1 (1)	< 1.20
activated Partial Thromboplastin Time (s),	27 (4)	27–39
Fibrinogen (mg/dL)	382 (122)	200–400
D-dimer (ng/mL) ^a^	1822 (1264)	< 500
Haemoglobin (g/dL)	11.8 (1.5)	12.0–15.0
Platelet count (×10^9/L)	209.6 (89.3)	172–440
Neutrophil cell count (%)	76.9 (7.6)	39.6–74.7
Lymphocyte count (%)	16.6 (7)	21.1–52.8
Neutrophil-lymphocyte ratio	4.7 (2.6)	< 4.5 (disease severity)
Lactate dehydrogenase (U/L)	210 (85)	84–246
Aspartate (U/L)	23 (14)	15–37
Alanine (U/L)	19 (11)	12–78
Bilirubin, (mg/dL)	0.14 (0.08)	0–0.20
Indirect	0.3 (0.2)	0–0.75
Total, median (IQR)	0.4 (0.3)	0.20–1.00
Creatinine (mg/dL)	0.6 (0.15)	0.51–0.95
hs_C reactive protein (mg/L)^b^	5.3 (0–165)	< 2.9

^a^ 83 women

^b^ 62 women

Pre-existing co-morbidities were recorded in four women (three with thyroid dysfunction, one with ulcerative colitis). At hospital admission, most women (63.3%) were asymptomatic for COVID-19 and 24.4% showed more than one sign or symptom of infection (Table 1). One woman (35 years, BMI 27, 41 weeks) needed Intensive Care Unit (ICU) for severe disease, another one (30 years, BMI 25, 32 weeks) required hospitalization for 1 month in the Infectious disease department.

With regard to inflammation markers, we observed NLR and hs-CRP above reference values in 49/90(56.6%) and 40/62 (65%), respectively (Table 2). Among those with NLR above 4.5, two showed respiratory symptoms and four had foetal demise (two losses in the first trimester and two IUFD). Furthermore, NLR was significantly different in nulliparae vs multiparae (median 5.4, IQR 2.9 and 4.5, IQR 1.6 respectively, Mann-Whitney U, p: 0.05).

Among those with hs-CRP above the reference value of 2.9 mg/L (*n* = 40), four showed respiratory symptoms and shortness of breath during hospitalization, three had gestational complications (gestational diabetes, PPROM and IUFD, respectively).

D-dimer measurement at admission was available for 83 women: median values were 1822 ng/ml (IQR 1264). Most cases (*n* = 70, 84.3%) showed values above 1000 ng/ml.

### Clinical course of the index pregnancy

**Fig. 1:**
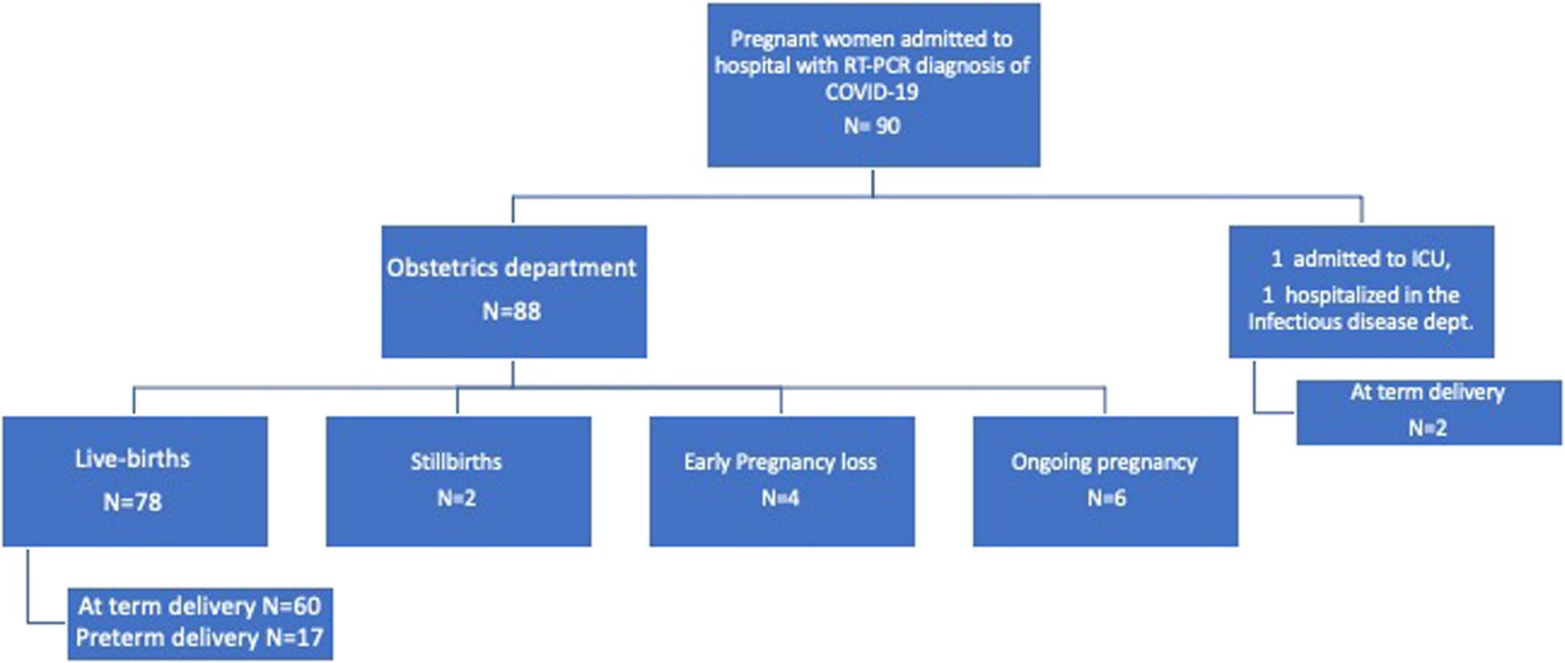
Flow-chart of the enrolled obstetric population

Figure 1 summarises outcomes of the index pregnancy: overall, 78 women had live births (21.5% of them with a pre-term delivery), two IUFD, four miscarriages, in six cases pregnancy was ongoing at the last day of follow-up (Fig. 1).

Table 3 shows pregnancy outcome and foetal and maternal characteristics. Overall, most women (43/78, 55.1%) had vaginal delivery (VD), 21.8% (17/78) had a preterm delivery. In six out of 17 preterm deliveries, women showed more than one respiratory sign or symptom. Five patients were administered with steroids, whereas in one case oxygen therapy was necessary. Furthermore, four pregnancies were complicated by gestational diabetes (in one case preeclampsia was also diagnosed and delivery occurred at 34 weeks) and one by HELLP syndrome.

**Table 3:** Pregnancy outcomes and feto-maternal characteristics ^a^

	Respiratory symptoms	Gestational age	Neonatal birth-weight	5-min Apgar score < 7
VD (*n* = 43)	9	39 (32–42)	3260 (2350–4050)	2
CS (*n* = 35)^b^	6	38 (32–41)	3155 (1990–4900)^c^	2
Miscarriage (*n* = 4)	0	10 (9–14)	/	/
IUFD (*n* = 2)	0	23 (21–25)	/	/
PPROM (*n* = 3)	0	40 (39–41)	3770 (3540–3830)	0
Preterm delivery (*n* = 17) ^b^	5	37 (32–37)	2895 (1990–4050)^c^	2

*VD* Vaginal Delivery, *CS* Caesarean Section, *IUFD* Intrauterine Foetal Death, P*PROM* Preterm Premature Rupture of Membranes

^a^ 6 ongoing pregnancies

^b^ Three twin pregnancies

^c^ Excluded twin pregnancies

### ADAMTS13 vonWillebrand factor axis

**Fig. 2:**
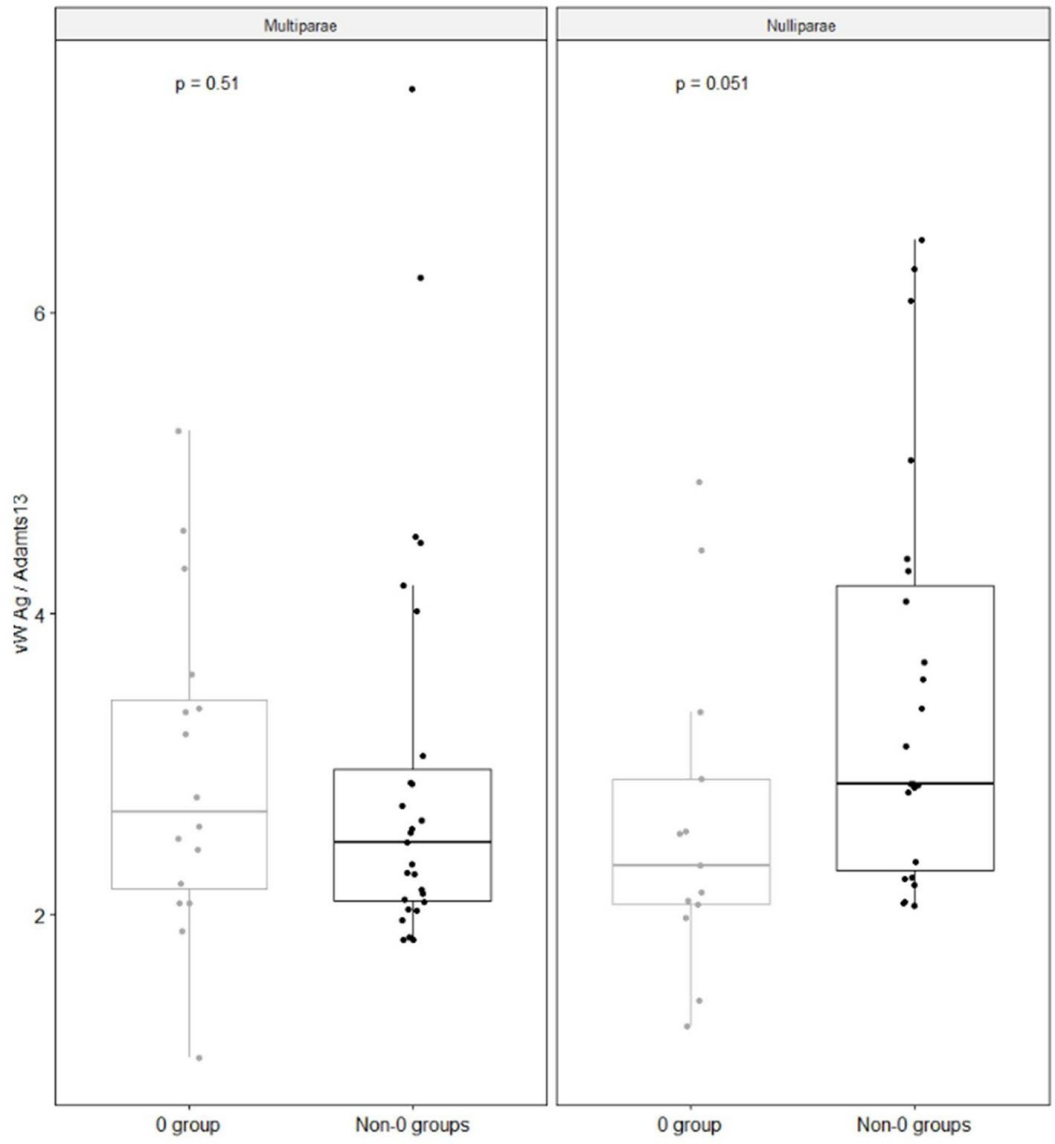
The figure shows vWF Ag/ADAMTS13 ratio according to parity and blood groups

**Fig. 3:**
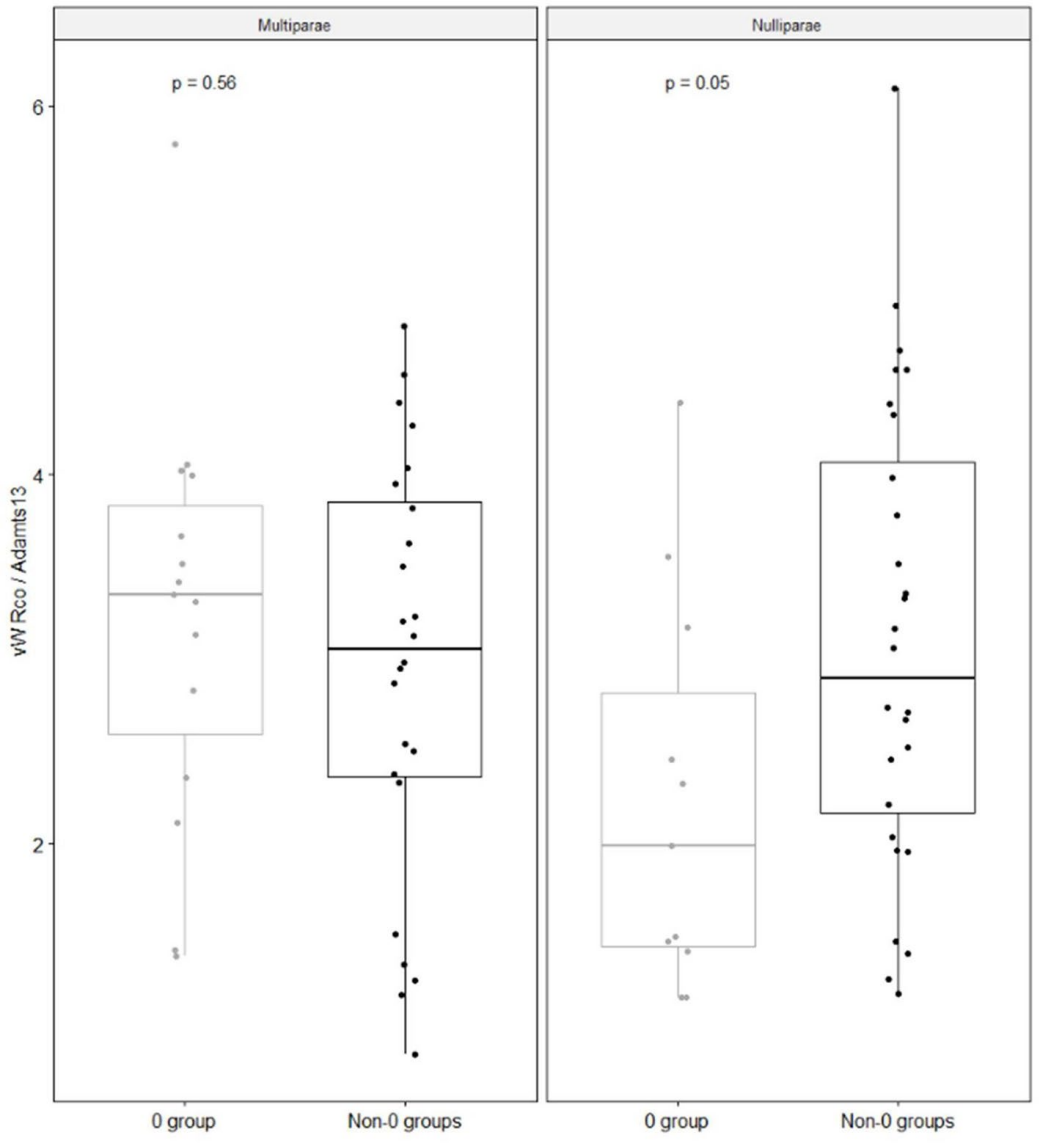
The figure shows vWF Rco/ADAMTS13 ratio according to parity and blood groups

ADAMTS-13 values, FVIII, vWFAg and Rco at admission are shown in Table 4. Differences between O and non-O groups were not significant, although a trend toward lower levels in FVIII and vWF was observed in O group. When we stratified our sample according to parity and group (O vs non-O), nulliparae with group O showed vWF Ag and Rco/ ADAMTS-13 ratios significantly lower than non-O, whereas in multiparae this difference was not observed (Figs. 2 and 3).

**Table 4:** FVIII and ADAMTS-13 vonWillebrand factor axis by blood groups

	All *N* = 90	Group 0 *N* = 32	Non-0 groups *N* = 58	*p*
ADAMTS-13 U/ml	100 (20)	100 (15)	110 (30)	ns
FVIII U/ml	194 (82)	189 (92)	203 (91)	ns
vW FAg U/ml	235 (168)	234 (130)	235 (213)	ns
vWF activity U/ml	287 (176)	282 (189)	310 (173)	ns
vWFAg/ADAMTS13 ratio	2.5 (1.4)	2.5 (0.9)	2.6 (1.6)	ns
vWF activity/ADAMTS13 ratio	3.1 (2.0)	3.1 (2.2)	3.0 (1.8)	ns

All values are expressed as median and IQR

Willebrand RCo values and its ratio with ADAMTS13 were significantly different in preterm deliveries vs at term ones (Mann-Withney U, *p* < 0.05). Logistic regression, correcting for age, BMI, parity, D-Dimer, NLR, comorbidities, FVIII, Willebrand Ag and RCo, blood group, haemoglobin values at admission, confirmed that ratio vWF to ADAMTS13 was significantly and independently associated with preterm delivery (OR 1.9, 95%CI 1.1–3.5).

## Discussion

### Main findings

In the present study, most pregnant women with a documented SARS-CoV2 infection were asymptomatic. With regard to foetal and maternal outcome, we found a significant higher prevalence of preterm births (21.5%) than that expected in general population (5–7%) [20]. It has been hypothesised that the imbalance of VWF and ADAMTS-13 in COVID-19 may promote multi-organ thrombosis with a clinical picture of thrombotic microangiopathy [21]. In adult non-pregnant patients with COVID-19, elevated VWF/ADAMTS-13 ratios were found associated with disease severity, being highest in those with worse illness or in non-survivors [21].

A novel finding of present study is that vWF to ADAMTS13 ratio is significantly and independently associated with preterm delivery, being the risk doubled for each increase of one unit in the ratio.

Another novel information is that group O nulliparae had vWF to ADAMTS-13 ratios significantly lower than non-O (Figs. 2 and 3), whereas this difference was not observed in women with at least one previous livebirth. It is known that ABH may influence susceptibility to Willebrand cleavage by ADAMTS-13 [22]. Individuals with blood group A and B show proteolytic cleavage by ADAMTS-13 lower than that of individuals with group O [23]. This depends on carbohydrate residues with terminal sialic acid residues, that are absent in type O individuals [22]. These findings are of clinical relevance, as variations in VWF sialylation have been described not only in patients with VW disease, but also in patients with a number of other physiologic and pathologic conditions. It has been observed that several pathogens, as *Streptococcus pneumoniae*, *Haemophilus influenzae*, and *Pseudomonas aeruginosa* can alter the expression of sialic acid on plasma derived vWF, thus impairing the process of glycosylation [23]. In addition, oestradiol circulating levels are significantly affected by parity, being higher in nulliparae than in parae [16]. Therefore, in our setting higher oestradiol levels, together with an impaired vWF glycosylation because of group O, might be responsible for lower circulating levels of vWF.

Clinical course of SARS-COV-2 infection during pregnancy is generally mild and the outcome of women is good or satisfactory [24, 25]. Most maternal infections occur during the third trimester and result in a small increase in hospital admission, as well as in admission to the ICU [26]. However, several maternal complications have been reported [24, 27, 28]: among them, preterm delivery have a prevalence ranging from 16 to 29.7% [27]. Consistent with previous studies, in our series we observed 21.8% of preterm deliveries. Furthermore, we found a small proportion of symptomatic women (*n* = 33, 36.7%); only 2 out of 90 (2.2%) needed a long hospital stay (one in Infectious disease department and one in ICU).

The “cytokine storm” characterizing SARS-COV-2 infection is known to induce endotheliitis and increases susceptibility to multiorgan thrombotic and microvascular injury. Therefore, it is not surprising that vWF /ADAMTS13 axis can be involved in the thrombotic microangiopathy observed during COVID-19 outside pregnancy [8, 17]. In adult non-pregnant patients, the more the ADAMTS13 Willebrand axis is impaired, the more severe is the disease [21]. In our series, pregnant women displayed high levels of hs-CRP, D-Dimer and NLR ratio, which is consistent with a high degree of inflammation and with findings from previous studies [29, 30]. It is likely that the degree of inflammation in pregnant women with COVID-19 is a driver of the preterm delivery.

These results are consistent with histological placental features. Indeed, placentae of women with SARS-CoV2 infection show an increased expression of vWf in the endothelium of decidua and chorionic villi, with the highest expression in the most severe cases [3]. These findings indicate that the placental endothelium of women with COVID-19 displays a characteristic frequently observed in “inflammation”, that is one of the pathogenetic mechanisms of preterm delivery [31]. Therefore, it is not surprising that vWF/ ADAMTS 13 axis can have a role in predicting preterm delivery in this setting.

In several countries, observational studies suggested that pre-existing maternal conditions, as obesity, age above 35 years, as well as gestational diabetes or gestational hypertension are associated with preterm delivery in COVID-19 [32]. In this relatively large sample of Italian women, we show that, independently of age, BMI, parity, comorbidities, inflammation markers, blood group, haemoglobin values at admission, a vWF to ADAMTS13 ratios are significantly associated with preterm delivery. Therefore, monitoring these biomarkers can be helpful in predicting the occurrence of such obstetric complication in pregnant women with COVID-19.

Specific drugs, as steroids, low-molecular -weight heparins, hydroxychloroquine are mostly used in pregnant women with COVID-19. However, there is still great uncertainty on the safety /efficacy profile of these medications during pregnancy [33]. Antenatal corticosteroids are recommended by professional societies when indicated for foetal lung maturation among women with COVID-19 [34].

Although published data do not suggest that pregnant women have an increased risk of thrombotic complications related to COVID-19 [35], nevertheless the prothrombotic state of pregnancy, further intensified by the prothrombotic phenotype typical of the disease [36], might justify an antithrombotic prophylaxis according to the severity of disease, the presence of comorbidities or other intercurrent conditions [33].

An individualized approach, based on specific biomarkers, could be helpful in predicting severity of the disease and, in turn, the most proper therapeutic strategy.

### Importance of present findings

These findings might support decision making process to manage and follow-up pregnancies in women with COVID-19. First, they suggest that vWF to ADAMTS13 ratio can be used as a tool of preterm delivery in pregnant women with SARS-CoV-2 infection. Second, a progressive increase of this ratio throughout pregnancy in COVID-19 patients, might be helpful in selecting patients who need special care and possibly most powerful therapeutic approaches. Third, in a scenario where pregnant women are still reluctant to get vaccine against COVID-19, the increasing awareness of the foeto-maternal risks due to infection, should encourage their utilization in any trimester of pregnancy and during lactation.

With regard to research implications, our findings warrant further investigation on larger samples with a more extended follow-up to ensure generalizability of the results.

### Strengths and limitations

To the best of our knowledge, this is the first study carried out in pregnant women with COVID-19 which has explored the VWF/ ADAMTS13 axis. The study suggests for the first time that monitoring vWF/ADAMTS13 ratio can be helpful to identify pregnancies at risk of preterm delivery.

The observational nature of the study without a control group may have affected the internal validity of the study. We cannot exclude that some flaws may adversely have affected data interpretation and the generalizability of findings. However, logistic regression allowed us to control for the confounders that previous studies had highlighted.

The limited period of observation is another limitation of our study: indeed, clinical follow-up was available for a very limited period of time (days of hospitalisation). Therefore, we cannot rule out different clinical maternal or foetal outcome in a longer frame-time. However, the most relevant finding highlighted in this paper is the preterm delivery; we paid attention to not look for associations with conditions that could have changed over a longer period of follow up.

Lastly, we did not analyse the vWF multimeric pattern in our patients. vWF multimers are relevant markers of endothelial damage, that have been hypothesised to drive micro-thrombosis in COVID-19 patients [37]. On the other hand, this analysis is not widely undertaken by routine laboratories, because of length of test and requirement of specialist equipment. Furthermore, this test suffers from lack of method standardisation, and often variable and subjective results [37]. If on the one hand, the multimers analysis would have allowed more speculations on the pathophysiological mechanisms of the disease, on the other hand it would not have added useful information for the practical management of pregnant women with COVID-19.

## Conclusions

In women with SARS-CoV-2 infection, an imbalance of vWF/ADAMTS13 occurs. The vWF ADAMTS13 axis is likely impaired because of endothelial dysfunction caused by the SARS- CoV-2 -induced activation of endothelial cells, leading to a significantly higher and independent risk of preterm delivery in this setting. Therefore, monitoring these biomarkers might support decision making process to manage and follow-up pregnancies in women with COVID-19.

## Data Availability

The datasets used and analysed during this study are available from the corresponding author on reasonable request. Access to anonymised data may be granted following review of the request. Exclusive use will be retained until the publication of major outputs.
